# Supplementary material for: Modeling protective meningococcal antibody responses and factors influencing antibody persistence following vaccination with MenAfriVac using machine learning
Source: PLoS One. 2025 May 14;20(5):e0323384. doi: 10.1371/journal.pone.0323384 (PMC12077764; doi:10.1371/journal.pone.0323384)
Supplement: S1 File — (DOCX) [file pone.0323384.s001.docx]

**Supplementary Information**

This document describes the formulation of the machine learning-based approach, feature engineering, and the features used in the long-term and short-term models.

## **Formulation of the machine learning approach from mixed effects model**

Following White et al’s mixed effects approach for immunogenicity kinetics following meningitis vaccine, the antibody level $Ab\left( t \right)$ at time *t* after administering the primary vaccine can be modeled as follows:

$$Ab\left( t \right)={Ab}_{0}e^{-r_{l}t}+\beta\left( \rho\frac{e^{-r_{s}t}-e^{-r_{a}t}}{r_{a}-r_{s}}+(1-\rho)\frac{e^{-r_{l}t}-e^{-r_{a}t}}{r_{a}-r_{l}} \right)$$

where ${Ab}_{0}$ is the initial antibody level and $r_{l}$, $r_{s}$, and $r_{a}$ are decay rates of different types of antibody secreting cells (long-lived, short-lived, and all combined) responsible for vaccine-induced immunity. The decay rates as well as the parameters $\rho$ and $\beta$ are subject-specific.

The above equation can be rearranged and simplified as follows:

$$Ab\left( t \right)=K_{l}e^{-r_{l}t}+K_{s}e^{-r_{s}t}+K_{a}e^{-r_{a}t}$$

where $K_{s}$ and $K_{a}$ are parameters function of the above-mentioned subject-specific parameters, hence are also subject-specific, while $K_{a}$ depends on ${Ab}_{0}$ in addition to the parameters.

If the subject-specific features are denoted by vector **x**, the antibody level can be modeled as an unknown function of $\mathbf{x}$ and${Ab}_{0}$.

$$Ab\left( t \right)=f(\mathbf{x,}{Ab}_{0})$$

While the above formulation is an abstraction of the prediction using short-term model presented in our work, this can be expanded to the antibody level after the secondary vaccination level as well. This pertains to the long-term modeling approach which may involve multiple doses of vaccination. If the secondary vaccine is administered after time $\tau$, the antibody level can be expressed as another function:

$$Ab\left( t \right)=g(\mathbf{x,}{Ab}_{0},\tau)$$

The proposed machine learning approach is essentially approximating the above functions based on the available data which includes antibody levels at different times, subject-specific demographic and vaccine-related information. Moreover, owing to the exponential relationship between the antibody level and time as seen above, we use logarithm of the antibody level in the proposed model and throughout our analysis to reflect its linear relationship with time.

## **Predictor variables**

While designing the models described in the previous section, we used the domain knowledge to derive the predictor variables (also known as input features). Some of the key aspects of deriving these variables from the dataset are as follows:

1.      Following White *et al*’s model, we used the log values of the antibody level, considering its exponential dependence on time. Another alternative was to take exponent of time as a feature while keeping the antibody level as is, however, this did not work well in our preliminary experiments; hence we limited our experiments to the first approach only. This was also justified by the empirical evidence of the antibody levels following a log-normal distribution, as shown by White *et al.*

2.      To address variability in number of vaccinations during the observation period $t$, we obtained derived features as needed instead of using the individual vaccine dose features. Some examples of original features associated with each dose are the type of the vaccine (PsA-TT or Hib-TT or something else), the dose amount, the time when it was administered (full list in Table S1).

For both models, we used a variable to represent the number of vaccines administered. We also use another variable to specify the number of PsA-TT vaccines. For the long-term model, we derived several features like the total dose of vaccines (both overall and PsA-TT only), the time of most recent vaccine, the mean administering time of vaccines (full list in Table S2)*.*

3.      We also used age at the time of the study and at the time of first vaccine as derived features. Finally, we used z-score as a derived feature (calculated from height and weight) for our model.

**Table S1: Original variables in the dataset and their statistics**

| **Variable** | **Statistics (n=3130)** |
| --- | --- |
| **Demographic variables** | |
| Age (days) | mean: 1169.5, SD: 2159.4, range: 97-10592 |
| Sex* | counts: (Male: 1641, Female: 1489) |
| Height (cm) | mean: 83.5, SD: 33.3, range: 50.6-192.0) |
| Weight (kg) | mean: 13.5, SD: 14.5, range: 4-88) |
|  | |
| **Vaccine-related variables** | |
| Visits/antibody readings | count: 16873 |
| rSBA readings | mean: 5327.5, SD: 16206.9, range: 2-524288 |
| ELISA readings* | mean:18.4, SD: 53.1 , range: 0.045-1616.4 |
| Number of vaccinations (per subject) | Single dose : 1613  2 doses: 576  3 doses: 931  4 doses: 10 |

*not used in the current study

**Table S2: Features for the long-term and short-term model**

| **Long -term model** | **Short-term model** |
| --- | --- |
| Time since primary vaccination | Time since last reading available |
| Antibody level during primary vaccination | Antibody level during last reading |
| Age at the time of primary vaccination | Age during last reading |
| Z-score (calculated from height and weight) | Z-score (calculated from height and weight) |
| Type of vaccines | Type of vaccines |
| Number of vaccines (could be more than 1) | Number of vaccines (could be either 0 or 1) |
| Dose of vaccines | Dose of vaccines |
| Time of last vaccination | Time of last vaccination |
| Mean of time of vaccinations | - |
| Number of PsA-TT vaccines | Number of PsA-TT vaccines |
| Dose of PsA-TT vaccines | Dose of PsA-TT vaccines |
| Time of last PsA-TT vaccination | Time of last PsA-TT vaccination |
| Mean of time of PsA-TT vaccinations | - |
