# Supplementary material for: Modeling protective meningococcal antibody responses and factors influencing antibody persistence following vaccination with MenAfriVac using machine learning
Source: PLoS One. 2025 May 14;20(5):e0323384. doi: 10.1371/journal.pone.0323384 (PMC12077764; doi:10.1371/journal.pone.0323384)
Supplement: Attachment — Submitted filename: Response to Reviewers.docx [file pone.0323384.s004.docx]

Dear Editor(s) and Reviewers,

Thank you so much for reviewing the manuscript and providing your valuable comments to improve the quality of the manuscript. We have addressed the comments and made appropriate edits in the manuscript. Our responses are shown below along with the comments from the reviewers (in red font).

----------------------------------------------------------------------------------------------------------------------------

Reviewer #1:

The manuscript “Modeling protective meningococcal antibody responses and factors influencing antibody persistence following vaccination with MenAfriVac® using machine learning” by Nasir et al. addresses the public health challenge posed by meningococcal meningitis in the meningitis belt region in Sub-Saharan Africa. The authors argue that despite the introduction of the MenAfriVac vaccine to eliminate cases of Neisseria meningitidis serogroup A (NmA) in this region, the duration of post-vaccination immunity and the necessity of booster doses remains unclear. To address this gap, the authors propose a long and short-term model that uses demographic, medical and antibody variables to predict NmA antibody responses, as well as vaccine’s half-life. They use Explainable Boosting Machines (EBM) to model the long and short-term models and later they benchmark these results with linear regression and decision trees, where the EBM model shows better performance.
The study is well motivated by the necessity of assessing post-vaccination immunity. It also proposes two interesting approaches (long and short-term) for predicting antibody kinetics.

However, the manuscript has in my opinion some shortcomings:
1. Major issues
1.1. Introduction
- The authors state the following: “Under the current study, we developed new computational models using machine learning techniques in order to improve the utility of modeling to inform decisions on PsA-TT scheduling and dosing”. It is unclear to me whether they refer to the approach/formulation of long and short-term models or rather to the EBM model. If they refer to the former, this is not really a new development of a computational model but a rather new formulation on how to predict antibody kinetics based on long and short term responses. If the authors refer to the latter, it should be stated more clearly that they have contributed to the development of the EBM tool.
We do not propose EBM model. We adopted this predictive machine learning algorithm for our application of predicting antibody levels with two different formulations of the problem and suitable feature engineering for them. As suggested, we replaced ‘new computational models’ with ‘new computational frameworks’ to better reflect the contribution in the revised manuscript.

1.2. Dataset
- The exact variables which are available are unclear. Some demographic information is mentioned in the text and the authors refer to the supplementary information for the reader to check information on vaccination data. However, no such information was found in the supplementary S1. Referring to other variables as “etc” in the main text is not sufficient if an explicit list is not available in the supplementary. Table S1 contains information on variables incorporated into the long and short-term models, but as demographic information is missing from that table, it is unclear which data is really available for the authors.

Provided the list of variables in Table S1.

- There is no description of the cohort on the observables from the data collected (i.e., distribution of demographic variables, vaccination doses, clinical data). Descriptive statistics of the study variables will benefit the understanding of the cohort’s characteristics.
Provided the descriptive statistics of variables in Table S1.

1.3. Modelling
General
-  The formal equations of the model in the supplementary material S1 are only referred to at the end of the short-term model description in the main text. Having the reference earlier (i.e., when describing the long-term model) could enhance clarity of the model formulation.

Agreed. We added a reference to the formal equations while describing the long-term model to motivate our formulation.

-  The authors model each antibody task independently. This might lead to the loss of shared information between the tasks.

This is a completely valid point and was taken into full consideration while conceptualization of the problem formulation. However, this was not feasible to model the task as time series forecasting with shared information across readings within a single subject due to the inconsistency in the time gap between events like vaccination and antibody readings. Moreover, since the proposed model does not require more than one previous reading, it has the additional benefit of real-world applicability at the onset of a new study.

- Antibody information often comes with a lower/upper threshold of the data. Is this the case here? If yes, the authors should consider censored regression using appropriate loss functions.

The lower limit of quantitation for the assay was 4. Anything below that was given the value of LLOQ/2, which equals 2. There was no upper limit.

While we did not use censored regression as the current study focused on antibody levels as an effect of vaccination which rarely involved the lower limits, but it would be interesting to explore this as future work, particularly if the dataset and goal of the modeling involve many values below the threshold.

Long-term modeling: 
- While figure 1 a-b illustrates well which antibody readings are used for the prediction task, the readability of the text could benefit from including the information that the model only uses the baseline antibody level and no further readings are included.

Thank you for the suggestion! We added a phrase in the caption of the figure 1a for long-term modeling and a similar phrase for its short-term counterpart to highlight this.

- The authors claim that “A model limitation is that the estimate could be less accurate for small values of as the model is unlikely to encounter such time ranges with much frequency.” This should follow from the aforementioned sample description with descriptive statistics.

The descriptive statistics for the time range have been included to support the claim.

Short-term modeling:
- The authors state that “Prior studies have shown that the antibody level has an exponential relationship with time.[11] As a result, limiting the training samples to shorter ranges of time makes the model less prone to error, particularly if multiple readings from the same subject are available.” I agree that shorter time-intervals might reduce the variance of a prediction task but I do not see why this should be, relatively, higher for exponential relationships if the applied model allows for non-linear relationships.

Even though the model allows for the nonlinear relationship, the presence of noise leads to imperfect estimation of the parameters inherent to the model that captures this relationship. Similar levels of error in this estimation would lead to larger errors for the long-term model than the short-term one as the multiplier to the exponent is higher for longer time duration.

- The authors state that “However, the short-term model can be affected by compounding error if only the baseline antibody level 0 is provided.” I agree with this statement. I was wondering if this is the case for the author’s data (e.g. due to missingness) and if yes, how often is it the case?

The statement was intended to suggest a real-world test case scenario instead of reflecting the data. If applied for a new study with only the baseline reading available, the short-term model might have compounding errors to predict antibody levels at multiple points of time in the future while using the predicted level at each point as baseline for the next prediction.

-For me the following formulation is unclear: “It is worth noting that the impact of the past vaccination history (before −1) is implicitly used by the model via the last antibody level −1, avoiding the need to explicitly include them, owing to the exponential modeling”. I agree that the use of the past vaccination is redundant as it is included in the last vaccination. However, it reads as if this fact is because of the exponential model - which is not clear to me.

We agree that it is not necessarily due to the exponential modeling. Hence, we removed the phrase attributing the behavior to the exponential modeling in the manuscript.

1.4. Regression
-  The authors state that missing values are omitted for both long-term and short-term models; however, they do not justify this decision. I think it would be important to assess whether the dataset used for model training (i.e., without missing values) is a representative subsample of the original data. If the missing values follow a certain pattern, the model’s results might be biased to the characteristics of the training data and not generalizable to the whole dataset.

The missing values primarily arose from subjects who did not follow up due to reasons beyond the scope of the study and quality/measurement issues in the titer assay for antibody readings. As none of these reasons may indicate any characteristics of immunogenicity, we did not further analyze the missingness of the data.

- It is mentioned that the logarithm of antibody titer level is used for modeling. I believe this should be incorporated into the supplementary, such that the model’s specification follows:

Agreed. We added a sentence to the supplementary mentioning this.

- It is mentioned that the dataset was divided into training and test sets only once, with a 80-20 split. However, it is not mentioned whether authors used an internal cross-validation on the training set for parameter tuning of the Explainable Boosting Machines (EBMs) model. I believe it is important to state how the EBM tuning was performed to enhance the reader's understanding on how the EBMs parameters were chosen.

We indeed tuned the hyperparameters of the EBM by 5-fold internal cross-validation for each instance of model training. Once the optimal hyperparameters were obtained, we trained the model on the entire training set using those parameters. This information has been added to the manuscript as follows: “ We conducted a 5-fold cross validation over the training data to obtain the optimal parameters for EBM and used them for finally training the model on the entire training set.”

-Furthermore, splitting the data only once results in having one realization for the evaluation metrics, which are random variables due to their dependence on the sample. The robustness of the results could be enhanced by including outer-cross-validation. As the authors currently split the data by 80% and 20%, an outer 5-fold cross validation might be advisable.

We completely agree that it would have been more robust to perform outer cross-validation with different training and test splits of the dataset. However, in the scope of the current study, we chose not to do so as it would be computationally expensive, especially due to having multiple proposed and baseline models. Nonetheless, we conducted a bootstrapping experiment with half-life estimation to evaluate its robustness in terms of 95% confidence intervals.

- The authors employ linear regression and decision trees as benchmark models. It is unclear to me why they did not consider using more complex models such as XGBoost or LightGBM as comparison models, as these are widely recognized for superior prediction accuracy over linear regression and decision trees. I believe it would be more interesting to compare the EBM to such more robust approaches to have a solid benchmark.

Thank you for the suggestion. We conducted new experiments with XGBoost and LightGBM as baselines and reported their performance in Table 2. We also added a few sentences in the Methods section (under Regression) and the Results section referring to these baseline models.

- Furthermore, as the authors motivate their model as being different to the exponential model, it would be interesting to see how the exponential model performs on their data.

While it would have been really interesting to see how the proposed model’s performance with White et al’s exponential model, there is a fundamental difference between these models in how each data point is constructed. Each exponential model considers a single subject as one sample and predicts the future antibody level using multiple past values, whereas in our framework one sample constitutes only one past reading to predict the future level. So, it would be unfair to compare the performances across these models.

1.5. Half-life estimation
- In this section, the authors mention that MenAfriVac as the first vaccination is the only one used for the half-life modeling. However, this decision is not further justified, so it is unclear to me whether this has a clinical reason or rather a reason from the structure of the dataset itself.

In the scope of MenAfriVac vaccine studies, the half-life of a vaccine is defined with respect to a single dose without any residual effect of a previous dose. This is why the half-life estimation analysis was limited to antibody levels following the first vaccination only before the next dose was administered.

- It is stated that individuals that do not reach the immunity threshold of 128 are excluded for the half-life analysis. As this means that the half life is only valid for individuals that reach this threshold, I suggest to mention that the half life is actually a half-life conditioned on a positive response to the vaccine.

This is a great suggestion.

We added the following to clarify this in the manuscript: “It should be noted that our definition of half-life is conditioned on a positive response to the vaccine. Hence if an individual had no recorded antibody levels above the threshold, the subject was excluded from the analysis.”

- The authors first speak about 99% Confidence Intervals and then about 95% Confidence intervals. Please clarify.

The mention of the 99% confidence interval was due to a typo. We have fixed it in the revised manuscript and thank the reviewer for finding it.

- What does it mean that they bootstrapped the samples  “and predicted their future antibody levels, incrementally adjusting the model by one day”?

We recognized that the phrase might be vague, and rephrased and clarified the statement as “predicted their future antibody levels one day at a time, incrementally applying the model for the next day using the previous day’s antibody level as an input.”
 
1.6. Results
- The authors evaluate and compare EBM against the benchmark models using the R-squared and RMSE metrics. They mention the following: “The EBM model performed better than both comparator regression models, both in long-term and short-term approaches, with respect to both metrics”. However, the evaluation of the model’s performance using both R-square and RMSE is redundant, as the R-Square is just a monotone transformation of the MSE. Thus, I would suggest only reporting one metric if the authors do not have a good reason to report both (in that case, I still suggest to report that both metrics favor the same models by construction). The statement “with respect to both metrics” is, however, in both cases misleading.

We fully agree with the point raised about the monotonic relationship between R-squared and RMSE. We reported both metrics as we wanted to capture the numeric range or errors (through RMSE) as well as the standardized goodness of fit (through R-squared). We now replaced the phrase “with respect to both metrics” by “as reflected in the reported metrics”. We also added a sentence in the methodology to enunciate that both metrics favor the same models by construction and provide the rationale behind using both.

- The authors make the following observation: “Finally, we found that using more samples resulted in the best performance, suggesting that more data might further improve our models in the future, irrespective of the study protocol”. I did not understand what is the purpose of testing the model with different amounts of observations, as it is well understood that using more available information will increase the model’s prediction accuracy.

The added training data in this experiment came from different vaccination study programs from different countries, while the test data was specific to only one such program. Differences in study protocols and inherent differences in the data in such scenario may not always translate into improved performance by introducing more data to training, hence we conducted this experiment. To emphasize this point we added the phrase “despite possible variability between training and test data “in the aforementioned statement in the manuscript.

1.7. Supplementary
- In the section “Formulation of the machine learning approach from mixed effects model”, there is a typo in “[...] while Kl depends on Ab0  , the index of K should be L and not I.

We agree. The index might have appeared differently due to font rendering mismatch between the standalone equations and the inline mathematical notations like the one mentioned in the comment. In the revised version, we made them consistent to avoid any confusion.

- It was not clear to me immediately whether the supplementary information on the model’s mathematical formulation refers to the long-term, short-term modeling, or a generalization of both models. I understand that is rather the latest, where depending on whether it is a long or short-term model, the term Abn will be different. However, I think that stating this explicitly and putting examples on how the formulation could change for the long and short-term model could enhance the reader’s understanding in this section.

The initial formulation applied directly to the short-term model and was later expanded for the long-term as well by introducing the case of secondary/multiple vaccines. We added the following to clarify this: “While the above formulation is an abstraction of the prediction using short-term model presented in our work, this can be expanded to the antibody level after the secondary vaccination level as well. This pertains to the long-term modeling approach which may involve multiple doses of vaccination. “

- The authors state: “Following White et al’s model, we used the log values of the antibody level, considering its exponential dependence on time. Another alternative was to take exponent of time as a feature while keeping the antibody level as is, however, this did not work well in our preliminary experiments; hence we limited our experiments to the first approach only.”. I think it could be interesting to explain further why it was that the exponential approach did not perform well in their model. This could improve the reader’s understanding on when to use log transformation or the exponential of time for modeling antibody responses.

While we did not have a formal error analysis, our decision to use log transformation of the antibody levels instead of exponential of time was justified by an empirical analysis conducted by White *el a*l, which found that the antibody levels follow a log-normal distribution across the population. Thus taking a log transformation of the target variable (antibody level) is more appropriate for standard regression techniques which often assume standard errors to be normally distributed. We included a line in the text to mention this.

- Throughout the feature engineering section, there are strange characters in the text. This persists with different pdf viewers.

This likely might be caused by inline mathematical symbols. We formatted them and tested the resulting pdf to ensure that there is no such rendering issues anymore.

- In the second point on the “Feature engineering” section, the authors mention that they created features using individual vaccine doses information. Specifically, they mention: “Some examples of original features associated with each dose are the type of the vaccine (PsA-TT or Hib-TT or something else), the dose amount, the time when it was administered, etc.”. I don’t think the authors should summarize which features were available for later feature creation with the term “etc”, as it is important for the reader to understand which variables were available to later create the new variables used for the models. Thus, I would suggest listing the full original features available.

The entire original variable list is provided now in Table S1.

- Later on, they mention: “For the long-term model, we derived several features like the total dose of vaccines (both overall and PsA-TT only), the time of the most recent vaccine, the mean administering time of vaccines, etc.”. Again, the term “etc” should not be used, but instead the authors could refer to Table S1 which lists the features used for both long and short-term models.

We have removed ‘etc’ and referred to the table of features (Table S2 in the updated version).

1.8. General style
-  Mathematical expressions and symbols within text should be formatted appropriately, as they look like it is not a formula but rather plain text.            
We formatted them using MS Word equation editor in a consistent manner now.

2. Minor issues

2.1. Introduction
- In the sentence: “While adults get infected, children and adolescents do so at much higher rates”, a citation is needed to complement the statement.

We added the following citation for this statement:

Soeters *et al*, Bacterial Meningitis Epidemiology in Five Countries in the Meningitis Belt of Sub-Saharan Africa, 2015–2017, *The Journal of Infectious Diseases*, Volume 220, Issue Supplement_4, 1 December 2019, Pages S165–S174.

- The term medical variables is too broad; the variables are described later in the paper but could be good to already give a brief overview in the introduction (also because it is mentioned that this is one of the main differences between the authors’ approach and White et al.).

Added some examples to highlight the additional variables used.

2.2. Half-life estimation
- The sentence “For these data points, we used the initial antibody level (0) as input if it was above the threshold. If not, we used the highest recorded antibody level for the individual. Individuals with no recorded antibody levels above the threshold were excluded from the analysis” is a bit confusing. Maybe it is good to explain that if an individual does not record antibody levels above the threshold at any time point, it is excluded from the analysis.

Rephrased the sentence for clarity.

-----------------------------------------------------------------------------------------------------------------------------

Reviewer #2: Major comments:
• Overall this is an interesting manuscript using innovative methods to estimate duration of protection of MenAfriVac, which is an important public health and policy question that has been previously answered but can benefit from being revisited with newer methods.

We sincerely thank the reviewer for their valuable comment and share the belief that newer and alternate approaches like ours can provide insights to many important public health questions.

More detailed comments:

Abstract:
• Is there an extra word in this sentence? “In the short-term model, we found moderately high performance (R-squared = 0.59) for out-of-training-data subjects and END even better performance (R squared = 0.83) in the long-term evaluation.”
We fixed the typo by removing the extra word.

Methods:
• In the half-life estimation methods, “If an individual had no recorded antibody levels above the threshold, the subject was excluded from the analysis.” Can you explain this a bit more? I would imagine that individuals that don’t respond to the vaccine would be important to include in calculating/estimating population half-life, as they don’t help increase population immunity. If vaccine non-responders are excluded, then the estimated half-lives only pertain to those individuals that respond to the vaccine, so might be then artificially high when thinking about population-wide immunity. Could non-responders be considered to contribute 0 time to the half-life calculations, as they spent no time above the immunity threshold?

In the scope of this work, the half-life is defined by conditioning on the positive responders only to empirically characterize durability of a successful vaccination; non-responses may be characterized using a different measure and is beyond the scope of the manuscript. We want to know – when a person is appropriately immunized, how long does that immunity last? For example, if 100 people are vaccinated and only 50% respond, the half-life calculated using the entire population would be artificially and substantially reduced and would not give us a true picture of how long protection lasts for those who did respond (and would not help us decide when to give a booster to them).

 If one wanted to estimate the decline in the prevalence of immunity in a population over time, then non-responders would need to be included. You could then estimate how long it would take to reach 50% of the population being non-immune, for instance.

We added clarification to our manuscript addressing this.

Discussion:
• It looks like there is a missing reference in the first paragraph.

We added the missing reference.

• WHO policy recommendations for the new lower-cost pentavalent conjugate vaccine are available at: Meningococcal vaccines: WHO position paper on the use of multivalent meningococcal conjugate vaccines in countries of the African meningitis belt, January 2024

Thanks for sharing the new policy updates. We cited it in the appropriate place in the revised manuscript.

• It would be interesting to also compare these estimated MenAfriVac duration results to estimates of duration of protection for other meningococcal conjugate vaccines.

The estimated duration is higher than most other vaccines, for which immunity wanes after 3-5 years, though this duration varies by serotype and by the protein used for conjugation. We added this to the revised manuscript with supporting references.
Figures
• Figure 4 – can the x-axis be labeled?

The x-axis in figure 4 (4a and 4b) has been labeled as ‘feature importance’ now.

-----------------------------------------------------------------------------------------------------------------------------

In the submission package, we have provided the revised (clean) manuscript and the supplementary information, along with their marked-up copies highlighting the changes made. We also added the figures as a zipped folder (Figure 4a and 4b files have been revised as per reviewer recommendations). In addition, we added a PDF version of the supplementary copy in case there is any issue rendering mathematical equations.

We appreciate your consideration of the revised manuscript for possible publication in PLOS One and look forward to hearing back from you.

Best regards,

Md Nasir

Dr. Md Nasir

Senior Research Scientist

AI for Good Research Lab, Microsoft

Email: mdnasir@microsoft.com

Postal address: 1 Microsoft Way, Redmond, WA, 98052, USA.
