# Supplementary material for: Modeling protective meningococcal antibody responses and factors influencing antibody persistence following vaccination with MenAfriVac using machine learning
Source: PLoS One. 2025 May 14;20(5):e0323384. doi: 10.1371/journal.pone.0323384 (PMC12077764; doi:10.1371/journal.pone.0323384)
Supplement: Attachment — Submitted filename: Response_to_Reviewers_auresp_2.docx [file pone.0323384.s005.docx]

Dear Editor(s) and Reviewers,

Thank you so much for reviewing the manuscript and providing your valuable comments to improve the quality of the manuscript. We have addressed the comments and made appropriate edits in the manuscript. Our responses are shown below along with the comments from the reviewers (in red font).

----------------------------------------------------------------------------------------------------------------------------

Reviewer #1: I appreciate the authors' detailed responses to my inquiries. Their revisions have effectively addressed all of my concerns and significantly improved the manuscript. I have only two minor suggestions remaining; once these are addressed, I believe the manuscript will be ready for publication.

Thank you so much for your feedback. We really appreciate your rigorous and insightful suggestions which we believe have improved the quality of the manuscript.

Minor Points

1)
Original Comment
Antibody information often comes with a lower/upper threshold of the data. Is this the case here? If yes, the authors should consider censored regression using appropriate loss functions.

Author’s Response
The lower limit of quantitation for the assay was 4. Anything below that was given the value of LLOQ/2, which equals 2. There was no upper limit. While we did not use censored regression as the current study focused on antibody levels as an effect of vaccination which rarely involved the lower limits, but it would be interesting to explore this as future work, particularly if the dataset and goal of the modeling involve many values below the threshold.

New Comment:
I agree with the authors that if censoring is limited, then using methods for censoring might not be necessary. However, I suggest to discuss this briefly in the paper and to mention the fraction of censored observations.

We added the following in the Dataset section: “The lower limit of quantitation (LLOQ) for the assay was 4, and values below this threshold were assigned LLOQ/2 (i.e., 2). In our dataset, less than 1**%** of observations were below the LLOQ.”

We also mentioned in the “Conclusion and Future Work” section: “Given the limited number of censored values, we did not apply censored regression techniques. However, future studies with a higher fraction of censored values may benefit from such approaches to account for measurement limitations more rigorously.”

2)
Original Comment
The authors state that “However, the short-term model can be affected by compounding error if only the baseline antibody level 0 is provided.” I agree with this statement. I was wondering if this is the case for the author’s data (e.g. due to missingness) and if yes, how often is it the case?

Author’s Response
The statement was intended to suggest a real-world test case scenario instead of reflecting the data. If applied for a new study with only the baseline reading available, the short-term model might have compounding errors to predict antibody levels at multiple points of time in the future while using the predicted level at each point as baseline for the next prediction.

New Comment:
I suggest to include this reasoning in order to avoid confusions.

We edited our initial statement to include this reasoning.

-----------------------------------------------------------------------------------------------------------------------------

Reviewer #3: The manuscript “Modeling protective meningococcal antibody responses and factors influencing antibody persistence following vaccination with MenAfriVac® using machine learning” explores the modeling of protective meningococcal antibody responses and factors influencing antibody persistence following PsA-TT (MenAfriVac) vaccination. The study employs machine learning techniques, specifically Explainable Boosting Machines (EBM), to develop both short-term and long-term predictive models. These models incorporate demographic, clinical, and serological data to estimate antibody kinetics and vaccine half-life. The work is well motivated, potentially being relevant in the context of optimizing vaccination strategies, well-structured and generally clear; however, I have some concerns and suggestions that, if addressed, could enhance the clarity, reproducibility, and overall transparency of the study.

We really appreciate your constructive comments. We have addressed them below and updated the manuscript accordingly.

Major concern:

- Authors should provide a more detailed description of the pre-processing steps applied to both predictive and outcome variables? While some information is provided in Supplementary 1, a more explicit explanation in the main text could improve clarity for the reader.

Specifically:
• Was batch correction applied to the outcome variable, given that measurements were obtained from different studies under potentially varying experimental conditions? If not, please clarify the choice

We added the following in the main text (in the “Regression” subsection): “Due to variations in the design of the original constituent studies—such as differences in the number of vaccinations and the timing of vaccine administration—we derived summary variables for the long-term model. For instance, we used the mean time of vaccination rather than individual vaccination time points.”

• Were predictive variables subjected to normalization, decorrelation or other pre-processing techniques? Since the performance of some models used for comparison may be sensitive to these pre-processing choices, further details would be beneficial.

Thank you for the suggestion of highlighting this. We indeed performed some preprocessing step for linear regression, only one of the baselines. We included the following in the text: “For linear regression, the predictor variables were normalized, and the categorical variables were encoded with one-hot encoding. EBM and the other three baseline models did not require any such preprocessing due to their scale-agnostic nature.”

Minor concerns:

• The manuscript compares the model proposed by White et al with Linear Regression, Regression Trees, LightGBM, and XGBoost. Could the authors elaborate on the rationale for selecting these specific models? For example, were other ensemble or deep learning methods considered? A brief justification of these choices would provide a better context.

Thank you for your thoughtful comment. We selected Linear Regression, Regression Trees, LightGBM, and XGBoost for comparison primarily due to their balance between predictive performance and interpretability. Given that our goal was to evaluate and compare model interpretability—particularly with the Explainable Boosting Machine (EBM)—we prioritized models that offer transparent or explainable mechanisms.

Linear Regression and Regression Trees are inherently interpretable and serve as useful baselines. LightGBM and XGBoost, while more complex, provide feature importance metrics and can be interpreted. Although we considered other ensemble and deep learning methods, we excluded them due to their limited interpretability in the context of this study’s objectives. We have clarified this rationale in the revised manuscript (subsection “Regression”).

• In the "Half-life estimation" section, the authors define a protective antibody threshold of 128 rSBA. Could they provide a reference or a more detailed explanation supporting this threshold? A brief discussion on why this value is considered appropriate would strengthen the validity of this assumption.

We thank the reviewer for this valuable comment. The rSBA threshold of ≥128 was chosen based on prior studies that used rSBA as a correlate of protection in the context of meningococcal conjugate vaccines. Specifically, Andrews et al. (2003) validated this threshold by comparing rSBA titers to protective levels measured by hSBA and found that an rSBA titer of ≥128 reliably predicts protection, despite being more conservative than hSBA-based thresholds. While rSBA titers in the 8–64 range were found to be equivocal, titers ≥128 consistently indicated protective immunity. We have added a citation and brief explanation to the manuscript to clarify this choice.

• The "Model Interpretation" section presents feature importance rankings in Figures 4a and 4b, limited to the top 15 features. Would it be possible to provide a table or supplementary plot including all feature importance values? This would enhance transparency and allow readers to assess the relative contributions of less influential variables.

Thank you for the insightful suggestion. While we agree having the ability to compare with all feature importance values would have been interesting, the issue of doing so lies in the fact that there are many pairwise interaction features (13*12/2=78 for long-term model) along with the original variables make feature set fairly large (78+13=91 features in total) to meaningfully make such comparison across all features. Also, the scores for relative less important features appeared tapering off (below 0.1) beyond top 10-15 and due to linear nature of the model, their relative importances are practically insignificant. As an example, from the modeling perspective, it does not matter much whether a less important feature’s importance score is 0.01 or 0.05, as it is not significant compared to top feature importance values either way. Hence, we restricted the visualization to 15 features to keep it concise and informative.

• In the "Discussion" section, the phrase "we describe the development and evaluation of a machine learning algorithm to characterize..." might be somewhat misleading, as the original model was developed by White et al. A more precise wording, emphasizing the application and evaluation rather than the development of the model itself, would be more appropriate.

While the statistical model was developed by While et al, we did not use it directly, we just derived the predictor variables and took inspiration from original statistical model to formulate a regression problem (in two alternative frameworks- long-term and short-term model) and used machine-learning based algorithms to solve the regression. This is in contrast with White et al’s model, which was aimed at fitting the data directly and extrapolation. Despite these fundamental differences, we do agree that rephrasing the sentence would be more appropriate. We made the following revision:” In this article, we describe the formulation and evaluation of a machine learning-based framework to characterize and predict the kinetics of serum antibody response to meningococcal serogroup A vaccination.”

• Supplementary 1: The subscripts a, l, and s are not clearly explained. A brief clarification would enhance readability.

Thank you for the valuable suggestion. We added the clarification: “where ${Ab}_{0}$ is the initial antibody level and $r_{l}$, $r_{s}$, and $r_{a}$ are decay rates of different types of antibody secreting cells (long-lived, short-lived, and all combined) responsible for vaccine-induced immunity. “

• Tables S1 and S2: Do these tables share the same legend? Please write a separate legend for each Table.

Thanks for catching this. The legends in Table S2 were dropped since they were not used in the text.

• In the "Regression" section, the manuscript states that the long-term model includes 39 variables, while the short-term model includes 14. However, these numbers do not align with those in Table S2. Could the authors clarify whether this discrepancy arises from interaction terms, dummy encoding of categorical variables or something else?

Yes, this was indeed due to dummy (one-hot) encoding of the categorical variables. To avoid confusion, we now updated the manuscript to report the number of original variables and later mention the one-hot encoding as preprocessing step.

-----------------------------------------------------------------------------------------------------------------------------

We appreciate your consideration of the revised manuscript for possible publication in PLOS One and look forward to hearing back from you.

Best regards,

Md Nasir

Dr. Md Nasir for the authors

Senior Research Scientist

AI for Good Research Lab, Microsoft

Email: mdnasir@microsoft.com

Postal address: 1 Microsoft Way, Redmond, WA, 98052, USA.
